# Perceived Partner’s Self-Control and Social Support Effects on Relationship Satisfaction in Couples Experiencing Infertility or Miscarriage: Dyadic Analyses

**DOI:** 10.3390/ijerph19041970

**Published:** 2022-02-10

**Authors:** Anna Wendołowska, Ewa Kiełek-Rataj, Alicja Kalus, Dorota Czyżowska

**Affiliations:** 1 Institute of Psychology, Jagiellonian University, 30-060 Krakow, Poland; anna.wendolowska@doctoral.uj.edu.pl (A.W.); d.czyzowska@uj.edu.pl (D.C.); 2 Institute of Psychology, University of Opole, 45-052 Opole, Poland; erataj@uni.opole.pl

**Keywords:** infertility, miscarriage, perceived partner’s control, marital satisfaction, coping strategies, actor-partner interdependence model

## Abstract

The process that infertile couples and those after a miscarriage go through is unpredictable and difficult to control; therefore, it is associated with a lowered sense of control for both partners. Uncontrolled stress creates a higher level of anxiety, which is associated not only with a lower quality of life but also with worse results from infertility treatment and higher risks of miscarriage. The aim of this study was to analyze the relationship between the partner’s perceived self-control and marital satisfaction in the context of the partners’ coping strategies. The actor-partner interdependence model was applied to 90 heterosexual married couples. Our results show that men who perceive their wives as being more self-controlled and women who are perceived by their husbands as being more self-controlled feel more satisfied in their relationships. The effect of a partner’s perceived self-control on satisfaction with the relationship was weaker when controlled for the length of marriage. It also appeared to be moderated through the spouses’ use of social support. We conclude that the effects of the partner’s perceived self-control and social support are strong for marital satisfaction in the context of infertility and miscarriage.

## 1. Introduction

Infertile couples and those who have experienced miscarriages struggle with many stressors that negatively affect the physical and mental health of both partners [1]. The process from suspecting infertility to receiving diagnosis and treatment often takes a long time and involves opposing emotions between hope and delusion, loss of control, lowering self-esteem, inability to plan for the future, and difficulties in social interactions [2]. The diagnosis of infertility and then the process of its treatment—medical procedures that often exceed the couple’s intimacy barrier [3]—are sources of many anxieties related to the feeling of guilt, shame, and fear of childlessness [4]. As a consequence, infertile couples experience elevated levels of anxiety and depression [5,6], which is associated with a lower quality of their life [7]. Similarly, the loss of a pregnancy can be a source of a serious crisis, evoking intense emotions related to the loss of a child and dreams of motherhood [8]. The treatment process often increases anxiety, helplessness, and a prolonged state of fear of another miscarriage [4,9] accompanied by anger, guilt, sadness [10], and depression [4,11], and the result is a reduced quality of life [12] and a higher risk of miscarriage [13,14]. Helplessness, insecurity, and anxiety accompanying both infertility and miscarriage may increase due to factors such as aging, long waiting times for conception, duration of pregnancy, no obvious pregnancy symptoms, number of miscarriages, and fertility problems in the nearest environment [8,15,16]. 

The sense of control is central to effective coping [17]. People who have a high level of perceived control are more competent at dealing with stressful situations than those who doubt their ability to influence events. Uncontrolled stress creates a higher level of anxiety than controlled stress, which in the case of couples that are struggling with infertility and have experienced a miscarriage may result in worse results from infertility treatment [7] and a higher risk of miscarriage [13,18]. The aim of our study was a dyadic analysis of the relationship between the partner’s perceived self-control, and marital satisfaction, in the context of the coping strategies used by partners. Various definitions of self-control can be found in the literature such as willpower, ego strength [19], suppression of impulses by effort [20], the ability to delay immediate gratification in order to obtain a better reward in the future [21], self-discipline [22], self-regulation [23], the tendency to adhere to social norms [24], or agreeableness in the context of interpersonal relations [25]. For the purposes of this study, we define self control on the basis of the Giessen Test author’s description of the scale, defined as diligence, adherence to rules, financial discipline, punctuality, love of truth, and care for order [19]. We did not find a similar approach that has been used in other studies; thus, our analyses complement the current knowledge about infertility and miscarriage.

### 1.1. Infertility and Its Importance for the Functioning of a Couple and the Quality of Their Relationship

According to WHO standards [26], infertility is the inability to become pregnant after a minimum of 12 months from having sexual intercourse without the use of measures that prevent conception. It is estimated that the problem of infertility affects about 8–12% of couples, and the scale of the phenomenon is increasing. Infertility is one of the most stressful and even traumatic life events [27,28,29,30] in every culture and society [1]. Reproductive problems, as a difficult experience for both partners, have been found to be more stressful for women, resulting in a decrease in their sense of wellbeing and a sense of their own femininity [31,32]. Women exhibit higher levels of depression, anxiety, and emotional distress than men in connection with experienced infertility [3,33,34]. The stress of infertility, however, is experienced by both spouses and is important for their marital satisfaction [35,36,37] and the quality of their relationships [38,39], both emotional and sexual [40,41]. Inability to have a child despite efforts may affect the emotional and mental state of partners [42]; induce feelings of guilt and isolation [43,44], sadness, and anxiety [5]; the frequent consequence of which is depression, which affects both women and men [45]. They can result in a deterioration of the relationship between partners but sometimes strengthen these relationships [46,47].

### 1.2. Experiencing a Miscarriage and Its Consequences for the Functioning of the Couple

Similarly to infertility, pregnancy loss is an obstetric or reproductive failure. The loss of a child in the prenatal period is also a highly stressful event for both parents [48,49], which may be treated as a trauma [48,49,50,51]. A UK study found that 25% of women who miscarried appeared to meet PTSD criteria. Women experiencing miscarriages feel powerless, isolated and lonely, and often blame themselves [52]; most often, they experience sadness but also clinical symptoms of depression and anxiety [53,54]. In total, 27% of women who lost pregnancy suffer psychiatric morbidity within 7 to 10 days after a miscarriage [55]. It is known that both women and men mourn the loss of a child and suffer from the loss of hope for the family’s enlargement [56,57]. The manner in which partners deal with this and the emotions that they experience as a result affect their mutual relations [58]. Losing a child in the prenatal period affects interpersonal relationships [50,59,60,61], decreases satisfaction with the relationship [62,63,64], the quality of sexual intercourse [60,61], and increases conflict [62,63] and tension in the family system [50], even in cases where pregnancy was unplanned [65]. 

Pregnancy loss does not always have only negative consequences for the relationship and may contribute to the feeling of greater strength and closeness between partners [66,67]. Qualitative research has indicated that the sense of commitment and turning to the partner as the primary source of support fosters the emotional wellbeing of the partners and increases the sense of strength and closeness in the relationship after experiencing a miscarriage [68].

Most studies have focused on the psychological and psychiatric effects of losing a child in the prenatal period for women [64,69,70], while few research works refer to men’s experiences in miscarriage [50,71,72,73]. This marked disproportion in research is due to the fact that miscarriage is treated as the case of a woman who has experienced it. However, it is indicated that men experience sadness, depression, anxiety, and stress, as well as a sense of guilt, anger, and loneliness [55,56,73], while it is claimed that they experience these feelings less intensively and for a shorter time [74]. However, there are also studies that show that the level of sadness experienced by men may be higher than for women [71,73]. Attention is drawn to the differences between men and women in the way they express sadness and the coping strategies used. It is believed that men do not express their sadness, which results from the lack of full recognition of the loss suffered by men, as well as social expectations that men are strong and should not express their emotions and responsibility resulting from being a support for their partner [57]. Women openly express their sadness and seek social support, while men experience sadness more internally and use more avoidance coping strategies [72].

### 1.3. Self-Control and Relationship Satisfaction

When looking for factors determining satisfaction with an intimate relationship, researchers have also considered the importance of self-control. Research indicates a relationship between self-control and the functioning of a relationship and partners’ wellbeing [75,76], partner satisfaction, and relationship quality [77]. People with a higher level of self-control are perceived by their partners as more sensitive and affectionate [78], trustworthy, and reliable [78,79]. The self-control of partners has been found to be related to constructive communication [80], dedication [81], or forgiveness [82], which may translate into a better quality relationship.

It can be concluded that people with a high level of self-control are more prone to behave in favor of a partner, especially in the face of a conflict of their own and partner’s interests and their attitude towards themselves and the partner’s needs [83]. People with high self-control are more likely to discuss problems, which, as can be expected, affects the way they are resolved and, in the long term, affects satisfaction with the relationship [80]. Research indicates that self-control increases the ability to restrain selfish impulses and increases the propensity to act in favor of relationships [76,84]. A relationship was found between self-control and an increased tendency towards constructive responses to the destructive behavior of a partner [75] and a greater willingness to forgive sins committed by a partner [82,85], as well as to sacrifice a partner or relationship [81,86]. It is worth noting, however, that there are also research results showing that people with a lower sense of self-control showed a greater willingness to make sacrifices for their relatives compared to people with high self-control [87]. Self-control promotes a person’s wellbeing, increasing life satisfaction, mental adjustment, relationship satisfaction, and dyadic adjustment [79,88,89]. Research has also shown that seeking a personal-relational balance can be a mechanism through which self-control can promote both personal and relational wellbeing [88].

In the infertility or miscarriage context, which are unexpected and unplanned stressors, partners usually have difficulty coping and maintaining or regaining control over their own life [89,90]. Future lifestyle choices or choices regarding parentage or childlessness no longer seem to be under their control [91], which relates to higher perceived stress and lower relationship satisfaction [92]. There is a great need to feel safe and in control of couples with reproductive challenges [93]. Infertile women often involve their spouses in the treatment process to feel that their partner is in control [18,94,95]. Many of them admit that their partner is their primary source of strength [96]. The emotional lability of the partners results in uncertainty and imbalance, making it difficult to provide support [8,97]. 

Perception of partner’s self-control is an indicator of his trustworthiness [79]. Partners with high self-control better tune their communication to their partner when disagreements or problems arise. Additionally, partners can “delegate” each other to self-control; therefore, having a partner with greater self-control enables greater outsourcing [77].

The question arises as to whether self-control determines the satisfaction of couples struggling with reproductive problems. It can be assumed that in difficult situations, self-control may be of particular importance, especially if coping with these difficulties requires greater self-discipline, as is the case with infertility and treatment attempts. Self-control in infertile couples trying to conceive can be associated with a greater chance of success, which can also translate into satisfaction with the relationship. It also appears that, in couples who experience miscarriage, which is a sudden and unpredictable event often leading to a feeling of losing control of their own lives, a sense of self-control can contribute to their satisfaction with the relationship.

Most studies at the dyadic level confirm the relationship between the level of self-control and self-satisfaction with the relationship [98,99,100,101]. Less frequently, a relationship between the self-control of partners and their mutual sense of relational satisfaction has been indicated [100,102,103]. Research by Zuo et al. [83] indicates, on the other hand, weaker and less obvious relationships between self-control and relationship satisfaction. Rather, they allow the conclusion that self-control does not play such an unequivocally positive role in relationship satisfaction and partner wellbeing as previously suggested, and the relationship between these variables may be more complex and moderated by other variables.

Dyadic studies, which are relatively sparse in the context of self-control and relationship satisfaction, make it possible to capture the interrelationships between the variables relating to the partners. It was assumed that the stress resulting from reproductive failures, including both infertility and the prenatal loss of a child, has a dyadic nature, and the way partners act is important for how this is experienced and dealt with. Therefore, it was concluded that the dyadic approach is more adequate than the previous more frequent focus on the individual characteristics of the partners. The actor-partner interdependence model (APIM) [104] was developed as a conceptual framework for collecting and analyzing dyadic data, which makes it possible to determine the interdependencies between the partners. Using APIM, we examine the impact of spouses’ self-control on their own satisfaction and mutual satisfaction with the relationship, while controlling the social support used by partners.

## 2. The Purpose of the Study

The aim of the study was to test the effect of the wife’s self-control and the husband’s self-control on their relationship satisfaction in the context of a miscarriage or infertility. It was decided to examine two groups of spouses who faced difficulties that may affect their satisfaction with the relationship. One group consisted of spouses who experienced the prenatal loss of a child, while the other group were spouses diagnosed with infertility. The common denominator for both studied groups was the experience of loss. In the case of spouses who lost a child in the prenatal period, this was the loss of a child who had already appeared physically, while in the group of spouses diagnosed with infertility, this was the loss of a child who did not exist physically and whom they desire or desired. Both prenatal loss and infertility are treated as reproductive failures. 

Close relationships are defined by the interdependence that is likely to result in significant correlations between the responses of both partners [105]. Analyzing dyadic data, therefore, requires special analytic approaches that properly account for the statistical interdependence between partners’ data. APIMs have been found to be very useful in the study of dyadic relationships. It allows testing the influence of a person’s own predictor on his or her own outcome variable, which is called the actor effect, and on the outcome variable of the partner, which is called the partner effect. These two effects can be measured as nonindependent two persons’ responses and are interpreted as controlling for the each other [106]. Using the APIM [107], both the effects of the wife’s and husband’s self-control on their satisfaction with the relationship (actor effect) and the effect of the partner’s self-control on the wife’s and husband’s perception on each other’s relationship satisfaction (partner effect) were tested. In this regard, the results were expected to confirm the following hypotheses.

**Hypothesis** **1** **(H1).** *Partners who perceive their spouses as being more self-controlled feel more satisfied with their relationships (actor effect).*

**Hypothesis** **2** **(H2).** *Partners who are perceived by their spouses as being more self-controlled feel more satisfied with their relationships (partner effect).*

When beginning the research, a question was additionally asked about the importance of coping strategies used for satisfaction with the relationship of spouses who experience infertility or miscarriage and the relationship between self-control, relationship satisfaction, and coping strategies.

**Hypothesis** **3** **(H3).** *Coping strategies used by spouses moderate the relationship between partners’ perceived self-control and relationship satisfaction.*

## 3. Materials and Methods

### 3.1. Participants

Fifty post-miscarriage and 40 infertile couples took part in the study (N = 180). The average age of post-miscarriage spouses was 36.02 (women: M = 35.12, SD = 7.55; men: M = 36.92, SD = 7.34), and for spouses with infertility, it was 35.82 (women: M = 34.70, SD = 7.88, men: M = 36.93, SD = 7.48). The average length of marriage for post-miscarriage spouses was 11 years (SD = 8.07), and for infertile ones, it was 9.23 years (SD = 6.81). There were 92% childless spouses in the group of infertile couples, and 74% of post-miscarriage spouses already had children. A total of 88% of this group had lost a child due to miscarriage, and 12% lost a child as a result of fetal death. Most spouses (82%) miscarriaged only once, and 74% had lost a child in the prenatal period more than a year before. Others had lost a child two (12%), three (4%), or four or more times (2%).

### 3.2. Procedures

The participants from Opolskie and Śląskie voivodeships in Poland were recruited at gynecological and obstetric wards. They were contacted in person, and two questionnaire packages were handed over in sealed envelopes. Completed questionnaires were collected at the next visit. A total of 103 pairs were tested, but 13 were excluded from the analysis due to the large amount of missing data. Informed consent was obtained from all participants. The study was conducted in accordance with the Helsinki Declaration. This study did not require a decision by the Research Ethics Committee as it was not a clinical experiment.

### 3.3. Measures

A demographic questionnaire was designed, which also included open-ended questions about infertility or miscarriage (e.g., “When was the last pregnancy lost? (a) less than 6 weeks ago; (b) 6 weeks to six months ago; (c) six months to a year ago (d) over a year ago”; “Has there been a ritual to bury the body of the child/child? (a) yes (b) no (c) in some cases yes, in others, no”).

The Marriage Success Scale, developed by Braun-Gałkowska [108], is composed of 46 statements and examines indicators of spouses’ marital satisfaction. The Cronbach’s alpha in our study was 0.76.

The Giessen Test [109] consists of 40 bipolar statements rated from −3 to +3, with 0 being the neutral value [19], that form six dimensions: social resonance, pliancy, control, depressiveness, openness, and social potency. It is used to examine one’s own self-image and the image of one’s other significant people (spouses, partners, etc.). 

For the purposes of this study, analyses limited to the respondent’s own control and partner’s perceived self-control scales are presented. The Cronbach’s alpha coefficient in our study was 0.51.

The Family Crisis Questionnaire (F-COPES; [110]) is a 30-item self-report questionnaire used to assess a family’s coping strategies. The respondents refer to the given statements by marking on a five-point Likert Scale from “I strongly disagree” to “I strongly agree.” The questionnaire consists of five subscales: acquiring social support, seeking spiritual support, mobilizing family to acquire and accept help, passive appraisal, and reframing. The Polish version of F-COPES was developed by Radochoński [111]. Cronbach’s alpha scores were 0.84, 0.67, 0.65, 0.68, and 0.60, respectively, for the subscales and 0.87 for the entire scale.

## 4. Analysis Strategies

The means with standard deviation were calculated for all variables. Pearson’s correlations were used to examine the intercorrelational matrix among variables, the *t*-test for paired samples was used to analyze differences between men and women, and the *t*-test for independent samples was used to test differences between the groups of infertile and post-miscarriage couples. Correlations for each variable between men and women assumed a lack of independence of the results in the dyads [112]. We expected significant correlations between partner’s self-control, coping strategies, and marital satisfaction. 

Taking into account the interdependence of the dyadic data, we used the actor-partner interdependence model (APIM) for the analysis [104]. All APIM analyses were performed as part of Structural Equation Modeling (SEM; [113]) using the lavaan package. To test the differences between sexes, the differences between the actor and partner effects of the spouses were calculated [114]. All other analyses were performed using IBM SPSS Statistics (Armonk, NY, USA) 24 statistical package delivered by Predictive Solutions (PS IMAGO PRO Academic package). All tests were performed at the 0.05 significance level. The hypothesized model was evaluated using goodness of fit indices that included the chi-square and the root mean square error of approximation (RMSEA; acceptable fit ≤0.08) [115].

## 5. Results

Means, standard deviations, and *t*-tests for independent groups, examining differences between groups of infertile spouses and those who experienced miscarriage, are presented in Table 1. Levene’s test showed the homogeneity of variance of the compared groups of spouses who experienced infertility or miscarriage (*p* = 0.096–0.918). The *t*-test for dependent groups revealed few significant differences between men and women. Men evaluated their wife’s self-control as higher than the inverse. Women sought social and spiritual support more often than men and more frequently mobilized family to acquire and accept help. The *t*-test for independent samples showed significant differences between the studied groups (Table 1). Men from the group of spouses after miscarriage had significantly lower scores in the perception of partner’s self-control than men from infertile marriages; they also less frequently used a spiritual support strategy. Infertile women used the reframing strategy more often and had a significantly higher level of marital satisfaction than women who had miscarried. There were no differences between men and women in both studied groups in the context of acquiring social support, but both men and women in the infertility group had significantly higher results in the application of seeking spiritual support compared to men and women who had experienced miscarriage.

Spouses’ results correlated significantly in terms of relationship satisfaction, social and spiritual support, mobilizing family to acquire and accept help, passive appraisal, and reframing but not in terms of respondents’ own and their partner’s perceived self-control (Table 2).

In both men and women, few weak and moderate correlations between the tested variables were observed. There was a link between women’s satisfaction and their own and their partner’s spiritual support, their own reframing, and their husband’s perception of their self-control. Men’s satisfaction did not corelate with any of the analyzed variables. Both spouses’ self-control scores correlated with each other’s perception of the partner’s self-control. Additionally, men’s own self-control correlated negatively with mobilizing family to acquire and accept help; their wife’s perceived self-control correlated with a reframing strategy and men’s passive appraisal.

### 5.1. The Spouses’ Perception of Their Partner’s Self-Control as a Predictor of Marital Satisfaction

**Figure 1:**
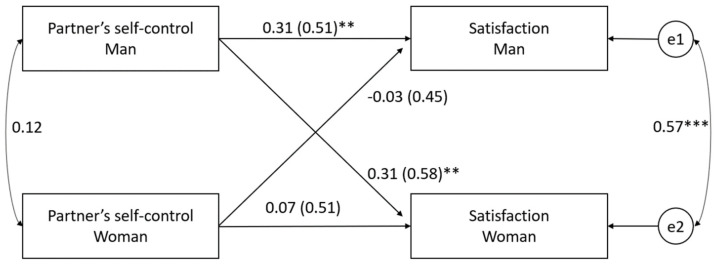
The partner’s perceived self-control and satisfaction with the relationship (Model 1). The independent and dependent variables are represented by rectangles; the two circles e1 and e2 present residual errors on satisfaction for males and females, respectively); the horizontal arrows describe the actor and diagonal arrows describe partner effects. The covariances between the independent variables are illustrated by curved double-headed arrows on the left; the curved double-headed arrow on the right represents the correlation between the two residuals; standardized coefficients are reported with standard error in parentheses. ** *p* < 0.01; *** *p* < 0.001.

The minimum sample size to detect the actor and partner effects for an APIM analysis given a desired level of power of 0.80 and alpha of 0.05 is 91 dyads [116], making our sample of 90 dyads acceptable for APIM analysis. The members of the dyad were distinguishable by sex (chi-square(12) = 25.52, *p* = 0.006). The independent variables and moderators were centered by subtracting the mean from all scores. Using the APIM (Table 3, Model 1), the relationship between the perceived partner’s self-control and satisfaction with the relationship was analyzed (Figure 1). The actor effect for men (1.54; *p* = 0.002, 95% CI (0.55, 2.54)) and the partner effect from men to women (1.81; *p* = 0.002, 95% CI (−1.02, 0.74)) was found to be statistically significant. Thus, it can be concluded that men who perceive their wives as being more self-controlled feel more satisfied with their relationships (H1 partially confirmed) and women who are perceived by their husbands as being more in control feel more satisfied in their relationships (H2 partially confirmed). 

In model 2, two independent variables were included in the analyses—the partner’s perceived self-control and respondent’s own self-control—in order to investigate the partners’ true characteristics (Table 3, Model 2). The results of the analyses show that the more partners idealize their wife/husband’s self-control, the more they are satisfied with the relationship, and the more the partners are idealized by their spouses as being self-controlled, the more they are satisfied in the relationship. With regard to the self-assessment of self-control, it can be observed that the sign of the relationship between the partner’s own self-control and satisfaction is negative, but both for the men and women, actor and partner effects are insignificant. The APIM with two independent variables (respondent’s self-control and partner’s perceived self-control) was tested simultaneously for the type of stress experienced by the spouses, i.e., infertility or miscarriage and the length of marriage (Table 3, Model 3). The satisfaction level of women who had had a miscarriage was found to be 9.31 points lower than in infertile women (*p* = 0.004), and in men, the difference in the satisfaction score was insignificant (4.92; *p* = 0.25). At the same time, the length of marriage appeared to weaken the relationship between perceived partner’s self-control and satisfaction with the relationship (−0.78, *p* < 0.001 for women and −0.80, *p* < 0.001 for men). The longer the duration of marriage, the weaker the effect of the partner’s perceived self-control on the spouses’ relationship satisfaction.

### 5.2. Moderating Effect of Acquiring Social Support on the Relationship between Partner’s Perceived Self-Control and Relationship Satisfaction

The degree to which the effects of a partner’s perceived self-control on relationship satisfaction were moderated by different coping strategies used by the spouses was investigated. Before conducting the analysis, the results of the independent variables and moderators were centered. Among all the coping strategies tested, only acquiring a social support strategy appeared to have a significant moderating effect on the relationship between a partner’s perceived self-control and relationship satisfaction.

In the moderation model with acquiring social support, the combined test of interaction for the two models was statistically significant (chi-square(4) = 9.87, *p* = 0.043, RMSEA = 0.128), which was sufficient evidence to state that there was a moderating effect of the coping strategy of acquiring social support. In the studied model (Table 4), we observe both significant actor and partner effects of a partner’s perceived self-control on relationship satisfaction. The effects for both the actor and partner of acquiring social support on satisfaction are insignificant; among interaction effects, actor-partner and partner-partner effects only were significant and were positive and negative, respectively. The effect of the spouses’ partner’s perceived self-control on their own relationship satisfaction increased as the partner’s acquiring social support increased. The effect of the partners’ perceived self-control on their spouse’s relationship satisfaction decreased as the partner’s tendency towards acquiring social support increased. A model in which constraints were placed on interaction effects indicated good fit indices (chi-square(1) = 0.04, *p* = 0.85, RMSEA = 0.00), which explained the pattern of interaction effects.

## 6. Discussion

Our results show that infertile and post-miscarriage women seek social and spiritual support and mobilize their families to obtain and accept help more often than men, which confirms the results of research showing that women more often seek support in stressful situations [117]. At the same time, research shows that men prefer problem-focused strategies [118]. In our research, women’s satisfaction correlated with their own and their partner’s spiritual support, their own reframing, and their husband’s perception of their wife’s self-control, which is consistent with the results of other studies. Loss of pregnancy and diagnosis of infertility are considered critical events that result in painful experiences, anxiety, and reduced quality of life [7,119]. Religion and spiritual beliefs have been recognized as resources used by infertile women to cope with suffering [120]. Spiritual support increases the coping capacity of couples experiencing infertility [121], and it is also very valuable after experiencing a miscarriage, where suffering and pain after the loss dominate. Religious feelings enable some bereaved women to cope better with the devastating effects of the loss of a baby through miscarriage [122], reducing the feeling of the irreversibility of death, explaining its meaning, and proposing rituals to help process the loss [123]. Similarly, reframing helps partners to redefine their traumatic experiences to make them more acceptable [124], both in the case of pregnancy loss [8,96] and infertility [117,125]. 

We observe a significantly lower level of satisfaction in post-miscarriage women than infertile women. At the same time, women who miscarried used the reframing strategy less frequently than infertile women, and both men and women in the miscarriage group had significantly lower results regarding the application of the strategy of seeking spiritual support compared to men and women who had experienced miscarriage. In the case of a loss of pregnancy, depressed mood, increased anxiety, and depression can last up to a year after the miscarriage [126], which certainly affects the mutual relations between partners [60,127] and translates into lowered satisfaction with marriage. Women facing miscarriage require social and emotional support [18,128]. The less frequent search for spiritual support by post-miscarriage couples might indicate their spiritual crisis and represent a threat to the very core of their beliefs about their sense of self, life, and ultimate truths [129], which at the same time may hinder the ability to engage in reframing, as this requires a radical reevaluation of one’s life goals [16]. 

In our study, men evaluated their wife’s self-control more highly than the inverse. Men’s wife’s perceived self-control correlated with her use of a reframing strategy and men’s passive appraisal. Additionally, men’s own self-control correlated negatively with their tendency to mobilize family to acquire and accept help. 

Self-control involves using a variety of coping strategies, which is also another method of reducing insecurity [130]. Using adequate coping strategies makes people feel more supported when dealing with stressful issues and prepares them to deal with problems more effectively [131]. The activity of women in the search for coping strategies, such as reformulation, coupled with the passive assessment of their husbands, is connected with the fact that they are perceived by their husbands as more self-controlled. Mobilizing the family to obtain and accept help in men seems to question their own image of self-control, which is confirmed by other studies showing that men tend to cope by distancing [89]; personal competences are more important for them in coping with stress than seeking support, and for women, the most important source of help is the spouse’s support [117] and support from other family members and friends [29]. 

Our results show that men who perceive their wives as being more self-controlled feel more satisfied in their relationships (H1 partially confirmed) and women who are perceived by their husbands as being more self-controlled feel more satisfied in their relationships (H2 partially confirmed). The longer the duration of the marriage, the weaker the effect of the partner’s perceived self-control on satisfaction with the relationship. The overall quality of life of infertile and post-miscarriage partners is significantly affected by the psychosocial impact of medical diagnosis and treatment course [132]. Marital distress arising from infertility or miscarriage, as well as unsuccessful treatment attempts, places couples at an even greater risk of anxiety and feelings of loss of control [133]. The ability to control has not only been identified as an important coping mechanism for infertility and miscarriage in women [90,134] but also in men [135]. Men may experience infertility and miscarriage indirectly through the impact that it has on their partners [1] by focusing mainly on her wellbeing [136]. For men, the stress associated with infertility or miscarriage can not only be eased through the couple supporting each other but receiving support from other sources also has a protective effect on marriage [137,138]. Social support from relatives, friends, and workmates has a beneficial effect on infertility and miscarriage stress [139]. Due to the fact that distress caused by infertility or miscarriage and the stability of the relationship seem to be intertwined concepts for men, it is possible that men feel that the burden of stress is lessened by knowing their partners are also being supported by other family members [29].

In the context of the analyzed specific stressors, such as infertility and miscarriage, female self-control seems to have a stronger influence on relationship satisfaction than male self-control. Experiencing fertility problems in marriage is a complex process that can trigger extreme emotions, guilt, and shame [140] is a common cause of low self-esteem in men [93] and often leads to frustration [94,141] and helplessness resulting from the feeling that the primary role of men is to support their partners [142]. Perhaps the role of men is limited to support, which on the one hand is very important for women in the context of infertility or miscarriage [96,143], but it is not a leading role from the point of view of reproductive success.

Additionally, most studies have focused on the psychological aspects of infertility and miscarriage in women [64,69,70], while only a few relate to men [71,72,73], which can also be interpreted as the fact that dealing with infertility mainly affects women, and men are not seen in the foreground in this situation.

Men in the post-miscarriage group perceive their wife’s self-control at a rate much lower than men in the group of infertile couples, which may be associated with the lower results for applying effective coping strategies such as reframing and spiritual support by women after a miscarriage compared to infertile women. Infertile couples and those after miscarriage use diverse coping strategies to handle their emotions, including searching for information, positively reappraising the situation, and seeking social and spiritual support [8]. It is possible that, when faced with the tragedy of pregnancy loss, wives’ coping strategies may not be effective enough to provide husbands with a sense of high self-control in a wife.

We assumed that spouses’ coping strategies would moderate the relationship between the partner’s perceived self-control and their relationship satisfaction (H3), which was partially confirmed.

The results indicate that the effect of the spouses’ partner’s perceived self-control on relationship satisfaction increases as the partner’s tendency to acquire social support increases. A partner actively seeking social support (a) strengthens the effect of their spouse’s perception of their self-control (the way the spouse perceives their partner in terms of self-control) on their spouse’s satisfaction and (b) weakens the effect of their perception of their spouse’s self-control (the way the spouse is perceived in terms of self-control) on their spouse’s satisfaction. In other words, the more the partner seeks social support, the more they are perceived by the spouse as having greater self-control, which translates into spouse’s satisfaction; at the same time, how the spouse is perceived in terms of self-control by the support-seeking partner becomes irrelevant. Social support is one of the mechanisms of resistance against the challenges of infertility [144] and miscarriage [18,128]. This can play an important role in reducing the negative effects of infertility and miscarriage-related stress and in ameliorating the effects of negative incidents, improving the couple’s self-control [8], self-confidence, and quality of life [145].

## 7. Conclusions

Among the strengths of our research that should be emphasized is the selection of the research groups of infertile couples and couples after pregnancy loss. Additionally, based on APIM analyses, it was possible to examine the effects of the perceived control of partners on their marital satisfaction by taking into account the social support sought by spouses [104].

Our research findings show a strong relationship between the partner’s perceived self-control and spouses’ marital satisfaction in infertile and post-miscarriage couples. At the same time, the results emphasize the importance of acquiring social support for both partners. Adequate social support is essential for infertile couples and those who have experienced a miscarriage to increase their wellbeing in both the short and long term [123,146]. The more satisfied women are with the level of support they receive, the less likely they are to experience mental health problems [147] but also the greater their chance of better fertility treatment outcomes and the lower their risk of further miscarriages [13,148].

Among the limitations of the presented study, its cross-sectional nature should be mentioned. Future longitudinal studies will allow for the in-depth analysis of the presented analyses, allowing the inference of the causal relationships between the analyzed variables. Another limitation is the relatively small group of respondents. The problem of infertility of miscarriage is a painful experience for couples and it is not easy to gather a larger group for analysis. For this reason, all conclusions drawn from the analyses should be treated with caution. The small size of the group also does not allow for separate analyses within both studied groups. Future research should also consider factors such as the reason for infertility/miscarriage, the number of previous miscarriages, or the duration of fertility treatment, which in turn could significantly influence the spouses’ perception of their partner’s control [117].

## Figures and Tables

**Table 1:** Descriptive statistics, genders, and differences between the infertility and miscarriage groups.

Variables	Infertility (n = 50)				Miscarriage (n = 40)				*t*-Test Men	*t*-Test Women	Men		Women		Sex Diff
	Men M/SD		Women M/SD		Men M/SD		Women M/SD				M	SD	M	SD	
Satisfaction	74.40	19.73	73.58	21.40	67.19	22.16	60.72	25.95	1.63	2.58 *	71.20	21.32	67.86	24.26	−1.57
Self-contr_O	27.18	3.76	28.24	3.85	27.33	3.72	24.43	4.25	−0.18	1.19	27.24	3.72	27.74	4.45	0.82
Self-contr_P	29.86	3.76	27.94	5.06	27.20	4.29	26.45	4.23	3.13 **	1.49	28.68	4.74	27.28	4.19	−2.24 *
Soc. support	27.38	5.38	30.34	5.80	26.53	5.96	29.63	5.53	0.71	0.59	27	5.63	30.02	5.66	4.36 ***
Spir. support	12.88	4.45	14.72	4	10.80	4.56	11.88	4.54	2.18 *	3.16 **	11.96	4.59	13.46	4.46	4.42 ***
Help accept.	15.16	3.29	16.84	3.20	15.43	2.48	16.02	3.83	−0.42	1.10	15.28	2.94	16.48	3.50	2.84 **
Pas. appraisal	7.86	3.01	7.54	2.48	8.35	2.28	8.00	2.67	−0.85	−0.85	8.08	2.71	7.74	2.56	−1.07
Reframing	26.90	4.16	27.12	5.24	26.38	3.51	24.43	4.25	0.64	2.63 *	25.67	3.87	27.92	4.98	−1.62

* *p* < 0.05; ** *p* < 0.01; *** *p* < 0.001. Self-control_O: own self-control; Self-control_P: perceived partner’s self-control.

**Table 2:** Pearson’s correlations between perceived partner’s self-control, coping strategies, and relationship satisfaction.

Variables		1	2	3	4	5	6	7	8	9	10	11	12	13	14	15	16
1	Satisfaction_A	1.00															
2	Social suport_A	0.14	1.00														
3	Spiritual suport_A	0.34 **	0.13	1.00													
4	Help acceptance_A	0.17	0.64 **	0.15	1.00												
5	Passive appraisal_A	−0.01	0.17	0.17	0.16	1.00											
6	Reframing_A	0.23 *	−0.02	0.36 **	0.00	0.28 **	1.00										
7	Self-control_O_A	−0.01	−0.09	0.07	−0.11	−0.22	0.12	1.00									
8	Self-control_P_A	0.11	0.10	0.11	0.17	−0.07	−0.17	−0.01	1.00								
9	Satisfaction_P	**0.61** **	0.13	0.19	0.18	−0.08	0.16	−0.08	0.01	1.00							
10	Social suport_P	0.11	**0.32** **	0.15	0.19	0.21	0.31 **	0.03	0.00	0.19	1.00						
11	Spiritual suport_P	0.33 **	−0.02	**0.75** **	−0.01	0.00	0.30 **	0.06	0.07	0.20	0.22 *	1.00					
12	Help acceptance_P	0.03	0.10	0.03	**0.23** *	−0.03	0.16	0.13	−0.03	0.18	0.50 **	0.06	1.00				
13	Passive appraisal_P	−0.03	−0.02	0.16	−0.02	**0.37** **	0.24 *	−0.12	−0.19	−0.06	0.24 *	0.00	0.00	1.00			
14	Reframing_P	0.20	−0.12	0.16	0.01	0.35 **	**0.54** **	0.01	−0.19	0.20	0.15	0.19	−0.03	0.33 **	1.00		
15	Self-control_O_P	−0.11	−0.01	−0.08	0.03	0.02	0.02	**0.02**	0.40 **	−0.13	0.10	−0.10	0.11	−0.24 *	−0.11	1.00	
16	Self-control_P_P	0.32 **	0.00	0.20	0.21	−0.05	0.25 *	0.30 **	**0.12**	0.30 **	0.14	0.18	0.20	−0.25 *	0.16	0.07	1.00

* *p* < 0.05; ** *p* < 0.01. (*n* = 90 dyads). _A women ratings; _P men ratings; Self-control _O: own self-control; Self-control_P: perceived partner’s self-control. Correlations between the dyad members are presented in bold along the diagonal.

**Table 3:** Actor and partner effects of perceived partner’s self-control on relationship satisfaction.

Model	Effect	Estimate	95% CI	*p*	Beta	*r*
Model 1	Women					
	Intercept	66.84	61.10 to 71.67	<0.001		
	Actor	0.35	−0.66 to 1.35	0.501	0.07	0.07
	Partner	1.81	0.68 to 2.95	0.002	0.31	0.31
	Men					
	Intercept	70.23	65.79 to 74.25	<0.001		
	Actor	1.54	0.55 to 2.54	0.002	0.31	0.31
	Partner	−0.14	−1.02 to 0.74	0.761	−0.03	−0.03
Model 2	Women					
	Self-control_P					
	Intercept	66.77	62.05 to 71.50	<0.001		
	Actor	0.70	−0.36 to 1.78	0.196	0.14	0.07
	Partner	2.03	0.87 to 3.20	<0.001	0.35	0.31
	Self-control_O					
	Intercept	66.77	62.05 to 71.50	<0.001		
	Actor	−0.59	−1.68 to 0.51	0.292	−0.11	−0.004
	Partner	−1.24	−2.59 to 0.12	0.074	−0.19	−0.11
	Men					
	Self-control_P					
	Intercept	69.98	65.87 to 74.08	<0.001		
	Actor	1.85	0.84 to 2.86	<0.001	0.37	0.31
	Partner	1.19	−0.81 to 1.05	0.804	0.03	−0.03
	Self-control_O					
	Intercept	69.98	65.87 to 74.08	<0.001		
	Actor	−0.93	−2.10 to 0.25	0.123	−0.16	−0.13
	Partner	−0.88	−1.82 to 0.07	0.070	0.08	
Model 3	Women					
	Self-control_P					
	Intercept	65.20	13.30 to 117.11	0.014		
	Actor	0.51	−0.52 to 1.55	0.332	0.1	0.07
	Partner	1.47	0.29 to 2.65	0.015	0.25	0.31
	Self-control_O					
	Intercept	65.20	13.30 to 117.11	0.014		
	Actor	−0.45	−1.50 to 0.60	0.398	−0.08	−0.004
	Partner	−1.05	−2.36 to 0.25	0.112	−0.19	−0.11
	Men					
	Self-control_P					
	Intercept	79.23	33.33 to 125.13	<0.001		
	Actor	−0.51	0.47 to 2.56	0.005	0.30	0.31
	Partner	0.02	−0.90 to 0.93	0.974	0.003	−0.03
	Self-control_O					
	Intercept	79.23	33.33 to 125.13	<0.001		
	Actor	−0.83	−1.98 to 0.33	0.159	−0.15	−0.13
	Partner	−0.76	−1.69 to 0.17	0.108	0.08	

**Table 4:** Effects in the acquiring social support moderation model.

Variables	Effect Type	Estimate	*p* Value	95% CI	Standardized
Self-control_P	Actor	0.87	0.01	0.17 to 1.57	0.25
	Partner	0.77	0.03	−0.31 to 1.46	0.20
Social support	Actor	0.39	0.15	−0.14 to 0.91	0.08
	Partner	0.21	0.43	−0.31 to 0.74	0.04
Interaction	Actor-Actor	−0.02	0.74	−0.13 to 0.09	−0.02
	Actor-Partner	0.13	0.03	0.25 to 0.15	0.15
	Partner-Actor	0.03	0.59	0.15 to 0.04	0.04
	Partner-Partner	−0.12	0.04	−0.01 to −0.14	−0.14

Note: Control_P: perceived partner’s self-control.

## Data Availability

The data that support the findings of this study are available from the corresponding author, upon reasonable request.
